# YeasTSS: an integrative web database of yeast transcription start sites

**DOI:** 10.1093/database/baz048

**Published:** 2019-04-26

**Authors:** Jonathan McMillan, Zhaolian Lu, Judith S Rodriguez, Tae-Hyuk Ahn, Zhenguo Lin

**Affiliations:** 1 Department of Biology, Saint Louis University, St. Louis, MO, USA; 2 Parks College of Engineering, Aviation and Technology, Program in Computer Engineering, Saint Louis University, St. Louis, MO, USA; 3 Program of Bioinformatics and Computational Biology, Saint Louis University, St. Louis, MO, USA; 4 Department of Computer Sciences, Saint Louis University, St. Louis, MO, USA

## Abstract

The transcription initiation landscape of eukaryotic genes is complex and highly dynamic. In eukaryotes, genes can generate multiple transcript variants that differ in 5′ boundaries due to usages of alternative transcription start sites (TSSs), and the abundance of transcript isoforms are highly variable. Due to a large number and complexity of the TSSs, it is not feasible to depict details of transcript initiation landscape of all genes using text-format genome annotation files. Therefore, it is necessary to provide data visualization of TSSs to represent quantitative TSS maps and the core promoters (CPs). In addition, the selection and activity of TSSs are influenced by various factors, such as transcription factors, chromatin remodeling and histone modifications. Thus, integration and visualization of functional genomic data related to these features could provide a better understanding of the gene promoter architecture and regulatory mechanism of transcription initiation. Yeast species play important roles for the research and human society, yet no database provides visualization and integration of functional genomic data in yeast. Here, we generated quantitative TSS maps for 12 important yeast species, inferred their CPs and built a public database, YeasTSS (www.yeastss.org). YeasTSS was designed as a central portal for visualization and integration of the TSS maps, CPs and functional genomic data related to transcription initiation in yeast. YeasTSS is expected to benefit the research community and public education for improving genome annotation, studies of promoter structure, regulated control of transcription initiation and inferring gene regulatory network.

## Introduction

Transcription initiation is the first and probably the most important step in gene expression, as it integrates the actions of key factors involved in transcription regulation (1). The short stretch of DNA immediately flanks the transcription start sites (TSSs), which is usually considered as the gene’s core promoter (CP), contain various sequence elements that accurately direct transcription initiation by the RNA polymerase II machinery (1). The accurate locations of TSSs and their transcriptional activities at a genome scale are invaluable for many studies. For examples, they can be used for precisely determining 5’ boundary and the 5’untranslated region (5’UTR) of protein-coding genes, improving genome annotation, inference the locations of CPs, identification of novel genes, CP elements and other motifs associated with transcription (2). In addition, because most genes have multiple CPs that have distinct activities in response to environmental cues or in different types of cells (2–5), quantitative TSS maps obtained from various growth conditions in a species are important for studying regulated control of transcription initiation and inferring gene regulatory network.

Many techniques have been applied to generate genome-wide TSS maps, such as microarray (6–8), serial analysis of gene expression (9), sequencing of full-length cDNA clones (10), RNA sequencing (11, 12), cap analysis gene expression (CAGE) technique (13, 14), transcript isoform sequencing (TIF-seq) (15) and transcription start site sequencing (TSS-seq) (16). Some of these techniques, such as CAGE, TIF-seq and TSS-seq, utilize `cap-trapping’ technology to pull down the 5′-complete cDNAs reversely transcribed from the captured transcripts. Through a massive parallel sequencing of the 5′ end of cDNA and analysis of the sequenced tags, the genome-wide TSSs can be identified at a single-nucleotide resolution (14). In addition, the number of mapped tags also provides a quantitative measure of transcript production from each TSS (14). CAGE is probably the most popular TSS interrogation technique and has been used to profile the locations and activities of TSSs in human (17), mouse, fruit fly and zebrafish (4, 13, 18) and the budding yeast *Saccharomyces cerevisiae* (2).

The high-resolution quantitative TSS maps in many organisms significantly improve our understanding of the complex and highly dynamic nature of transcription initiation landscape in eukaryotes (2–5). These studies revealed that most eukaryotic genes contain multiple CPs that are differentially used among different tissues or developmental stages or in response to environmental cues (2, 3, 5, 17). The selection and activity of different CPs of a gene are precisely regulated to ensure that a correct transcript is produced at an appropriate level in different tissues, developmental stages or growth conditions. It has been shown that misregulation of transcription initiation can cause a broad range of human diseases, such as breast cancer, diabetes, kidney failure and Alzheimer’s disease (19–23). For instance, the tumor repressor gene BRCA1 has two TSSs, which produce longer and shorter transcripts. The longer BRCA1 transcript is only present in breast cancer tissues, resulted in significantly reduced production of protein products due to 10-fold less of translation efficiency than the shorter transcript (22). Unlike the translational process, which starts at the same methionine codon AUG, transcriptions are initiated from many nearby nucleotide positions in most CPs. The transcription activities of different TSSs within a CP could be very different. Based on the distributions of TSS activities, the CPs can be classified to different shape groups, which have been shown to be related to different regulatory activities (3). In addition, it was found that transcription initiation is pervasive, and it can be initiated from unconventional sites, such as exons (2, 3), which generate transcripts that truncate or eliminate the predicted protein product.

Transcription initiation of eukaryotic genes is a complex and highly regulated process. Transcription of protein-coding genes is carried out by RNA polymerase II (Pol II). The recruitment of eukaryotic Pol II to a CP is facilitated by general transcription factors. The most extensively studied CP element in eukaryotes is TATA box, which is mostly found 25–30 bps upstream from the TSS in mammals. However, the location of TATA box in *S. cerevisiae* promoter was found located in a wide range of 40–120 bp upstream of TSS (24). Gene-specific transcription factors may function as activators and repressors to further increase or reduce the transcription level by binding to gene-specific transcription factor binding sites (TFBSs). TFBSs are highly enriched around 115 bp upstream of the TSS in *S. cerevisiae* (25). The presence of nucleosome in the promoter regions may be an obstacle for transcription initiation. Transcription initiation typically occurs near the boundaries of nucleosome-free regions (NFRs) (8). Several studies have found that genes with different expression profiles are associated with distinct nucleosome occupancy (NO) patterns in the promoter regions (26, 27). The promoters of constantly expressed genes usually contain a nucleosome-depleted region (NDR) where most TFBs are located (28, 29). In contrast, conditionally expressed genes, such as stress-response genes, are associated with nucleosome-occupied promoters (27). The activation of transcription is also accompanied by alteration of chromatin structure, such as ATP-dependent nucleosome sliding (30, 31) or histone modifications (HMs) (32). Methylation of lysine residues in histone H3 could increase transcription by weakening chemical attractions between histone and DNA and enable the DNA to uncoil from nucleosomes (32).

**Table 1.** Yeast species, genome assembly and CAGE data

Species	Strain	Assembly	CAGE reads	No. of TSS (>0.1 TPM)	No. of CPs
*C. albicans*	SC5314	ASM18296v3	62 917 157	354 135	27 068
*Kluyveromyces lactis*	NRRL Y-1140	ASM251v1	59 908 113	377 665	23 163
*Lachancea waltii*	NCYC 2644	ASM16711v1	47 907 986	312 918	24 069
*Lachancea kluyveri*	NRRL Y-12651	ASM14922v1	36 988 291	220 879	17 732
*Naumovozyma castellii*	CBS 4309	ASM23734v1	58 897 449	276 990	19 382
*Saccharomyces bayanus*	623-6C	ASM16703v1	30 739 852	288 198	19 617
*S. cerevisiae*	S288c	R64–2-1 (SacCer3)	37 200 564	324 133	24 033
*Saccharomyces mikatae*	IFO 1815	ASM16697v1	63 468 342	246 308	17 109
*S. paradoxus*	CBS432	ASM207905v1	45 578 830	427 801	30 419
*Schizosaccharomyces japonicus*	yFS275	SJ5	52 907 395	318 951	25 536
*Sch. pombe*	972 h-	ASM294v2	60 584 140	264 007	24 093
*Yarrowia lipolytica*	CLIB122	ASM252v1	48 428 838	356 722	28 307

**Table 2.** Resources and databases used for compilation and curation of data in YeasTSS

Data type	Data track	Species	Data description	Data format	Data source
TSS	YPD	All species	TSS maps produced by nAnT-iCAGE grown in YPD	BW	This study
	Cell arrest; DNA damage; diauxic shift; glactose (2%); glucose (16%); H_2_O_2_; heat shock; NaCl	*S. cerevisiae*	TSS maps produced by nAnT-iCAGE from cells growth in eight other conditions		(2)
	Pelechano_2013_YPD; Pelechano_2013_Galactose	*S. cerevisiae*	TSS map generated by TIF-seq from cells grown in YPD and Galactose	BW	(15)
	Malabat_2015_YPD	*S. cerevisiae*	TSS map generated by transcription start site sequencing in wild type yeast cells grown in YPD	BW	(16)
	Arribere_2013_YPD_Plus; Arribere_2013_YPD_Minus	*S. cerevisiae*	TSS identified by TL-seq	BW	(41)
	Doris_2018_TSS-seq	*S. cerevisiae*	TSS maps obtained by TSS-seq from wild-type and spt6 mutant strain	BW	(42)
	Li_2015_CAGE	*Sch. pombe*	TSS identified by CAGE in YPD	BW	(44)
	Thodberg_2019_CAGE	*Sch. pombe*	TSS maps by CAGE from different grown environments	BW	(45)
CP	YPD	All species	CPs inferred based on nAnT-iCAGE TSS maps from cells grown in YPD	BED	This study
	Consensus; cell arrest; DNA damage; diauxic shift; galactose (2%); glucose (16%); H_2_O_2_; heat shock; NaCl	*S. cerevisiae*	CPs inferred from nAnT-iCAGE TSS maps from cells growth in eight other conditions	BW	(2)
TATA-box	Rhee_2012	*S. cerevisiae*	TATA-box or TATA-like elements identified based on ChIP-exo data	GFF3	(52)
TFBSs	Venters_2011_25°C; Venters_2011_37°C	*S. cerevisiae*	TFBS based on ChIP-chip data with a 5% FDR threshold for cells grown at 25°C and at 37°C	GFF3	(50)
	MacIsaac_2006	*S. cerevisiae*	Refined TFBS map based on re-analysis ChIP-chip assays	GFF3	(48)
	Wood_2012	*Sch. pombe*	Predicted TFBS by Pombase	BED	(51)
RNA polymerase II binding	Ghavi-Helm_2008_WT_RNA_PolII_YPD_16°C; Ghavi-Helm_2008_WT_RNA_PolII_YPD_30°C;	*S. cerevisiae*	Genome-wide location of RNA Pol II based on ChIP-chip assays	BW	(53)
HMs	Kirmizis_2007_H3K4me1; Kirmizis_2007_H3K4me2; Kirmizis_2007_H3R2me2a; Kirmizis_2007_H3K4me3	*S. cerevisiae*	H3K4me1, H3K4me2, H3R2me2a, H3K4me3 based on ChIP-chip assays	BW	(55)
	Pokholok_2005_H3K14ac_vs_H3_H2O2; Pokholok_2005_H3K14ac_vs_H3_YPD; Pokholok_2005_H3K36me3_vs_H3_YPD; Pokholok_2005_H3K4me1_vs_H3_YPD; Pokholok_2005_H3K4me2_vs_H3_YPD; Pokholok_2005_H3K4me3_vs_H3_YPD; Pokholok_2005_H3K9ac_vs_H3_YPD; Pokholok_2005_H4ac_vs_H3_H2O2; Pokholok_2005_H4ac_vs_H3_YPD;	*S. cerevisiae*	The distribution of methylated and acetylated histones based on ChIP-chip assays	BW	(56)
NO	Field_2009_YPD; Field_2009_GAL	*S. cerevisiae*	*In vivo* nucleosomes occupancy for *S. cerevisiae* cells grown in YPD and galactose	BW	(46)
	Field_2009	*C. albicans*	*In vivo* nucleosomes occupancy for *C. albicans* cells grown in YPD	BW	(46)
	Lantermann_2010	*Sch. pombe*	*In vivo* nucleosomes occupancy for *Sch. pombe* cells grown in YPD	BW	(47)
DS features	Zhou_2013	*S. cerevisiae*	Computation of DS features including minor groove width, roll, propeller twists and helix twists	BW	(54)
TBs	Waern_2013_AlphaFactor; Waern_2013_Benomyl; Waern_2013_Calcofluor; Waern_2013_CongoRed; Waern_2013_DNADamage; Waern_2013_GrapeJuice; Waern_2013_HeatShock; Waern_2013_HighCalcium; Waern_2013_Hydroxyurea; Waern_2013_LowNitrogen; Waern_2013_LowPhosphate; Waern_2013_OxidativeStress; Waern_2013_Salt; Waern_2013_ScGlycerolMedia; Waern_2013_ScMedia; Waern_2013_Sorbitol; Waern_2013_StationaryPhase	*S. cerevisiae*	Transcript coordination obtained in 18 growth conditions based on RNA-seq	GFF3	(12)

To obtain genes’ TSS or 5’ boundary information, most researchers rely on genome annotation files conforming to the GTF (general transfer format) or GFF (general feature format). Although a few alternative TSSs may be provided in the genome annotation files, it is not feasible for the annotation files to include the detailed information of all TSSs, such as the number and activities of TSSs within and a CP, regulated activities of CPs in different cell types or under different growth conditions and CP shape. Therefore, it is necessary to visualize the TSS maps to obtain a more intuitive, accurate, informative picture of transcription initiation landscape. In addition, due to the complexity of transcription initiation in eukaryotic cells, integration and visualization of functional genomic data related to transcription initiations to the TSS maps provides an intuitive illustration of the relationships between the structural components and functional elements, which could facilitate future studies about regulatory mechanism of transcription initiation. The visualization also serves as useful tools for teaching and public education to demonstrate the complexity and dynamics of transcription initiation of eukaryotic genes.

The human and mouse TSS maps can be visualized through `The FANTOM web resource’ (33) and `ZENBU’ (34). ZENBU also provides data integration, data analysis and visualization system enhanced for RNA-seq, Chip-Seq and other types of high-throughput data (34). The *Saccharomyces* Genome Database (SGD) has been a popular resource for yeast research community that provides integration and visualization of various functional genomic data (35). To our knowledge, there is no web resource dedicated for visualization and integration of functional genomics data related to the transcription initiation landscape for the yeast species. Many yeast species have been served as important model organisms, widely used in daily human life, as workhorse for food, brewery and energy industries. Particularly, the budding yeast *S. cerevisiae* has served as eukaryotic model organisms for many landmark discoveries in gene regulation mechanisms and other cellular processes over the past several decades (36). In this study, we generated high-resolution TSS maps for 12 important yeast species with divergence times ranging from 5 to 300 million years (37). We inferred CPs for each of these species based on the quantitative TSS maps. We built a new web database: YeasTSS (www.yeastss.org), to provide free access, visualization and integration of the TSS maps, predicted CPs, as well as other functional genomic data related to transcription initiation for yeast species. This depth and breadth of this database are valuable for the research community to improve the genome annotation of these yeast species, to study the regulated and evolutionary dynamic of transcription initiation landscape and its underlying genomic changes.

## Materials and methods

### Generation of TSS maps and inferences of CPs

TSS maps and inferred CPs are the core data of YeasTSS. We generated the TSS maps for the 12 yeast species using a revised CAGE protocol called no-amplification non-tagging CAGE libraries for Illumina sequencers (nAnT-iCAGE) (38). The nAnT-iCAGE protocol does not involve PCR amplification or restriction enzyme digestion (38), which reduced the bias created by the two processes. The cells of each species were inoculated and grown to mid-log phase in yeast extract–peptone–dextrose (YPD) medium at 30°C for total RNA isolation. Two biological replicates of nAnT-iCAGE libraries were generated for each species. All nAnT-iCAGE libraries were sequenced using Illumina NextSeq 500 (single-end, 75 bp reads) at the DNAFORM, Yokohama, Japan. A total number of 838 624 674 reads were generated from the 24 libraries (Table 1). The raw CAGE sequencing data have been deposited in the NCBI Sequence Read Archive (SRA; Bioproject accession number: PRJNA510689).

We identified TSS and infer CP based on the nAnT-iCAGE sequencing reads using a protocol described in (2) with necessary modifications. Briefly, CAGE tags were mapped to the reference genome of each species using HISAT2 (39) with no soft-clipping allowed. If the annotation of rRNA is available, CAGE tags mapped to rRNA were removed using rRNAdust (http://fantom.gsc.riken.jp/5/sstar/Protocols:rRNAdust). Only those uniquely mapped tags, (621401708) were used for TSS identification. CAGE-detected TSS (CTSS) were defined after correcting systematic G nucleotide addition bias at the 5’ end of CAGE tags. To filter background noise, we only considered those CTSSs with a tag per million (TPM) of ≥0.1 for CP identification. A CP defined in this study is a cluster of nearby TSSs in a small genomic region, which reflect the transcriptional activity from the same set of general transcriptional machinery. Specifically, TSSs within 20 bp of each other were clustered as tag clusters (TCs). We then calculated the distribution of CAGE signals within a TC and used the positions of 10% quantiles and positions of 90% quantiles of CAGE signals as the boundaries of this TC. If the boundaries of two TCs are <50 bp, TCs were then aggregated together into a single-set non-overlapping consensus clusters, corresponding putative CPs.

The quantitative TSS maps and CPs of *S. cerevisiae* in nine growth conditions were obtained from our previous study (2). These condition-specific TSS maps were generated using *S. cerevisiae* BY4741 strain (*MATa his3Δ1 leu2Δ0 met15Δ0 ura3Δ0*).

### Sources of genome sequence, annotation and functional genomic data

The reference genome sequence and the annotation files were obtained from the SGD (35), NCBI Genome or Yeast Gene Order Browser (40) (Table 1). In addition to the TSS maps generated in this study, YeasTSS also include 5’ boundary of transcripts data of *S. cerevisiae* (15, 16, 41–43) and Schizosaccharomyces *pombe* (44, 45) obtained from other studies. We integrated multiple functional genomics data related to transcription initiation regulation and promoter structures in several well-studied species (Table 2). The *in vivo* NO data of *S. cerevisiae* and *Candida albicans* were downloaded from Field *et al*. (46). We obtained the *in vivo* NO data of *Sch. pombe* from Lantermann *et al*. (47). The locations of TFBS locations in *S. cerevisiae* were determined according to the motif-discovery algorithm from MacIsaac *et al.* (48), which is based on reanalysis of ChIP-chip data (49), and from Venters *et al*. (50). The TFBS data in *Sch. pombe* was retrieved from (51). The lists of TATA box motif in *S. cerevisiae* were obtained from (52). YeasTSS also integrates the genome-wide location information of RNA polymerase II, which were obtained by ChIP-chip assays (53), DNA shape (DS) features (54), HMs (55, 56) and RNA transcript boundaries (TBs) inferred from RNA-seq data (12) in *S. cerevisiae*. The types of functional genomic data, description of data and their source used in YeasTSS are shown in Table 2.

### Data format

BED (Browser Extensible Data) format was used for functional genomic data only requires genomic coordinate information, such as the predicted CPs, TFBS locations, TATA box and TBs. BedGraph is a simple tab-delimited text format that is popular on most genome browser including the University of California Santa Cruz browser (57) for displaying the numerical value of the sequencing data at each position. However, the BedGraph text format is slow for loading and refreshing of a large data set in most genome browsers. BigWig (BW) format is a compressed binary format designed for displaying high-throughput next-generation sequencing data efficiently and effectively in genome browsers (58). We used BW format for data including both genomic coordinates and signal strength, such as quantitative TSS maps, HMs and NO. The `BigWig’ files were converted from `BedGraph’ format to allow fast and efficient remote access to the web browser server from end-users’ computers. The genomic sequence, annotation and functional genomic data in each yeast species were integrated by their genomic coordinates.

### Database development, implementation and genome browser configuration

The YeasTSS web resource was built using various computing techniques, such as Amazon Web Service (AWS), Apache web server, JBrowse utilities, PHP, Python and Bootstrap framework. Instead of using a SQL or non-SQL database, we used various data types and in-house programs for retrieving and searching data in fast turn-around time that reduced the burden of data management and save time and costs by migrating data files to the database. The web server was built on Amazon Elastic Compute Cloud (Amazon EC2) that is an AWS web service to provide secure and resizable compute capacity in the cloud. The web server stores all data files and relevant programs for the YeasTSS database. Apache HTTP Server (Version 2.4.34) is used as a web server engine. JBrowse (Version 1.14.0), a genome browser being developed as the successor to GBrowse (59), was selected and incorporated into YeasTSS by its fast, scalable and usable advantages. JBrowse provides fast and smooth scrolling and zooming to explore genome and sequencing information. It can scale easily to multi-gigabase genomes and deep-coverage sequencing data. JBrowse also supports various formats of sequencing data including GFF3, BED, FASTA, Wiggle, BigWig, BAM, VCF, REST and more. Especially, BAM, BigWig and VCF data can be displayed directly from the compressed binary file with no conversion or extraction needed. The search functionality of the database uses PHP (Version 7.0.30) to parse the query terms and Python (Version 3.6.5) to retrieve data from the database and returns outputs in a user-friendly format. The Bootstrap (Version 4.1.1), an open source toolkit for developing with HTML, CSS and JavaScript, is used in the YeasTSS web pages.

## Results

### YeasTSS web interface

**Figure 1:**
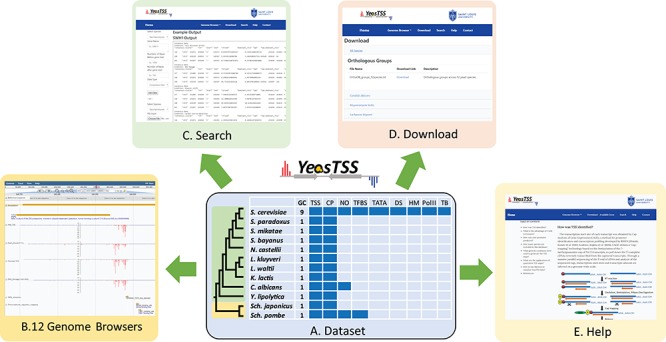
The overall design of YeasTSS. (**A**) Dataset: The dataset used in YeasTSS is illustrated in this central table. Currently, YeasTSS includes 12 yeast species. The evolutionary relationships of these species are demonstrated by the phylogenetic tree on the left side of data table. The clade of 10 budding yeast species is shaded in green, and the clade of 2 fission yeast species is shaded in yellow. The CP and TSS data of each species were generated by this study. NO data are integrated for *S. cerevisiae*, *Sch. pombe* and *C. albicans*. TFBSs are available in *S. cerevisiae* and *Sch. pombe*. For *S*. cerevisiae, several other functional genomic data are also integrated: TATA-box, DS, HMs, Polymerase II binding (PolII) and TBs obtained from 18 different growth conditions. (**B**) Genome browser: These data are visualized and integrated by dedicated JBrowse genome browser of each species. (**C**) Search: The `Search’ utility provides search tools in to retrieve TSS and CP information from gene-by-gene analysis or global approaches. (**D**) Download: The `Download’ utility allows users to download all raw data used in this database through web interface. (**E**) Help: The `Help’ page provides documentations about of CAGE technique, TSS identification, inferences of CPs and instructions of using genome browsers.

YeasTSS (http://www.yeastss.org/) aims to provide visualization, access and integration of quantitation TSS maps, CPs and various functional genomic data related to transcription initiation for important yeast species. YeasTSS provides free access and is not password-protected, and it does not require login or registration. As of December 2018, YeasTSS contains 12 different yeast species (Table 1 and Figure 1), including 10 budding yeast species (such as model organism *S. cerevisiae* and human pathogen *C. albicans*) and two fission yeast species (including another model organism *Sch. pombe*). The evolutionary relationships of these species, which were obtained from previous studies (37, 60), were shown by the phylogenetic tree in Figure 1. The divergence times among these species ranges from a few million years (between *S. cerevisiae* to *Saccharomyces paradoxus*) to over 300 million years (between fission yeast and budding yeast) (37). The wide range of divergence times is valuable for studies of the evolutionary dynamic of transcription initiation landscape of promoter structures at different time scales.

**Figure 2:**
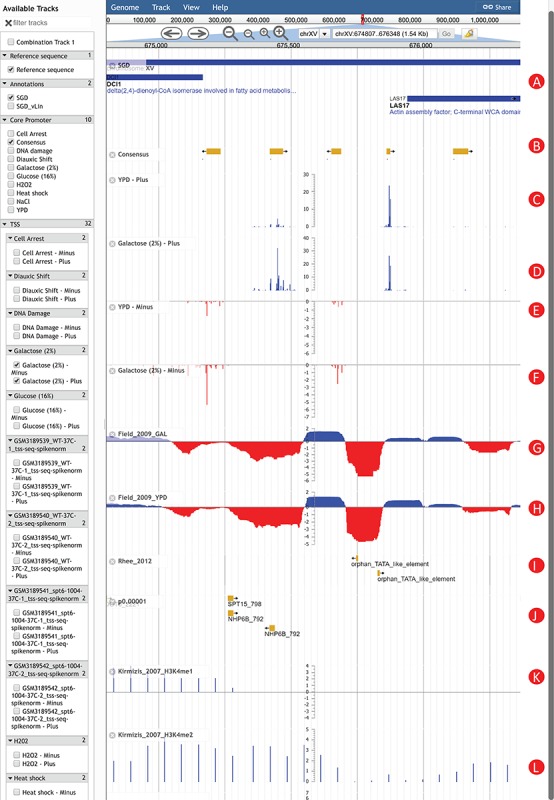
An Example of using YeasTSS Genome Browser to explore transcription initiation landscape. A 1.5 kb region around the CP region of LAS17 on chrXV (674 807–676 348). The available tracks in *S. cerevisiae* are provided in the left panel of genome browser. Two consensus CPs on the forward strand are present within 1000 bp upstream of LAS17 ORF. The transcription activity of each CP can be visualized by the TSS tracks. Different CP activities can be observed between the YPD and YPGal (galactose 2%) growth conditions in *S. cerevisiae*. Only a few tracks were selected in this case. These tracks include: (**A**) Genome annotation from SGD; (**B**) consensus CPs; (**C**) TSS map on plus strand under YPD condition; (**D**) TSS map on plus strand under YPGal (galactose 2%) condition; (**E**) TSS map on minus strand under YPD condition; (**F**) TSS map on minus strand under YPGal condition; (**G**) nucleosome occupancy under YPD condition; (**H**) Nucleosome occupancy under YPGal condition; (**I**) TATA box; (**J**) position weight matrix (PWM) predicted binding sites; (**K**) histone modification H3K4me1; (**L**) histone modification H3K4me2.

The homepage of YeasTSS provides a brief introduction of the database and navigation to its three major utilities: `Genome Browser’, `Search’ and `Download’ (Fig. 1). These utilities can be accessed from the header bar of the homepage. The header bar is also available at all other pages of the website so users can access and jump to any data set from any page on the website. Under the header bar of the homepage, a phylogenetic tree shows the evolutionary relationships of included yeast species. By clicking the species name in the phylogenetic tree, it will open its own dedicated genome browser page in a new window. Alternatively, the users can click the species name from the dropdown menu from `Genome Browser’ in the header bar to open a new window for its genome browser. A brief description of the YeasTSS is also provided in the homepage. Detailed documentation of YeasTSS is provided by `Help’ webpage, which can be accessed from the link in the header bar. The `Help’ page provides information about of CAGE technique, TSS identification, inferences of CPs and instructions of using genome browsers.

### Data visualization and integration using JBrowse genome browser

Graphical web visualization and integration of TSS maps with other functional genomic data are carried out by JBrowse genome browser in YeasTSS. Each species has a dedicated JBrowse genome browser page. Users can open a genome browser for any species in a new window by clicking the species name in the phylogenetic tree at the homepage or by clicking the species name in the dropdown menu of `Genome Browser’ in the header bar. In the genome browser of each species, a user may click the chromosome names to switch the display between different chromosomes and use the left and right arrows to browse different chromosome regions. To visualize the transcription initiation landscape or promoter structure of a gene or a specific genomic region, users can enter the chromosomal coordinate ranges, gene or locus name in the search box of the genome browser. JBrowse includes a `faceted’ track selector, which allows users to interactively search for the tracks they are interested in based on the metadata for each track. By clicking on the features of non-quantitative tracks, such as gene annotation, predicted CP, TFBS or TATA box, users will be brought to the detail description page of the features and its associated nucleotide sequences.

To illustrate how YeasTSS can be used to visualize the complex and dynamic transcription initiation landscape and promoter structure, we demonstrated an example of using YeasTSS JBrowse genome browser to explore the *LAS17* (YOR181W) gene in *S. cerevisiae* gene (Fig. 2). *LAS17* encodes an actin-binding protein involved in actin filament organization and polymerization (61). To simplify the illustration, only a few tracks in faceted track selector were selected. As shown in Figure 2, two predicted CPs, which are about 500 bp apart, are present in the upstream of *LAS17* ORF, suggesting that two different sets of transcription machinery may be assembled to initiate the transcription of *LAS17*. Here, we called the CP that is more proximal to the *LAS17* ORF as `LAS17-CP1’, while the distal one as `LAS17-CP2’. Transcription from the two CPs generates transcripts isoforms with distinct lengths in the 5′ untranslated region (5’ UTR). The presence of multiple CPs in a gene has been shown to be prevalent in *S. cerevisiae* (2) as well as in mammals (3).

The detailed transcription activity within a CP is visualized by the TSS track, which is shown as XY-Plot wiggle track. The transcription activity from each TSS is normalized as TPM. The TPM values on the *y*-axis scale of XY-Plot wiggle track are auto-scaled to the range of TPMs in the displayed window. The TSS signals of the forward (plus) strand and reverse (minus) strand are shown in separate tracks for better visualization effects, considering the TSS signal strengths could be dramatically different between the two strands in some genomic region. The TSS signals of the forward (plus) strand are shown as positive TPM values, while TPM values are negative from the reverse (minus) strand. Within a CP, transcription is initiated from a cluster of nearby TSSs, rather than a single TSS, which generates transcripts with slightly different lengths. A dominant TSS that accounts for the majority of transcriptional activities is found in most CPs. The distributions of TSS activity signals are very different among CPs, forming different promoter shapes, ranging from sharp to broad.

It has been found that CP shapes are associated with distinct regulatory patterns (3, 4). In this case, LAS17-CP1 demonstrates a sharper shape than LAS17-CP2. Interestingly, the transcription activities of LAS17-CP1 are much stronger than that of LAS17-CP2 grown in rich medium (YPD). However, the relative transcription activities from the two CPs have a significant shift if the glucose in the growth medium is replaced by 2% galactose. This phenomenon is called CP shift, which is found prevalent in yeast (2), mammals and fruit fly (62, 63). CP shift generates transcripts with distinct 5’ UTRs or results in usage of alternative translation start codon, which was speculated to have significant impacts on the translation efficiency (64, 65) or mRNA stability (66). According to the in vivo NO measured in yeast cells grown in YPD and galactose, both CPs are located near the 3’ end of NDRs, which is consistent to the genome-wide observations (8). Different H3 methylation patterns are shown near the CP of LAS17. For instance, strong H3K4me1 signals are detected in the entire ORF of LAS17, while H3K4me2 signals are enriched in the 5’ end of ORF. In contrast, H3K4me3 signals are mainly detected upstream of LAS17 ORF, corresponding to the promoter regions, suggesting that different histone methylations might occur at different regions of a gene. In addition, two putative TATA-box motifs are present near 5’ end of LAS17-CP1, while several TFBS motifs are found in the proximal region of LAS17-CP2, suggesting that probably different regulatory mechanisms were involved in the usage of the two CPs.

### Search and download utilities of the YeasTSS

The `Search’ utility provides search tools to retrieve TSS and CP information from gene-by-gene analysis or global approaches. The users have two ways to receive the CP and TSS data by using the search utility. First, a user can simply select a species and provide gene name(s) in the search box. A user may define the genomic range by providing specific numbers of base pairs before and after the start position of gene’s ORF (default values: 1000 bp upstream before gene start and 100 bp after gene start). Alternatively, a user can provide a gene list in comma-separated value format that should have a format of gene name, bases before gene start and bases after gene start position. Instead of traditional SQL-based database query, this program-based search is very fast and scalable. The search output includes the genome coordinates, transcription abundance of CPs (TPM) or tag counts of TSS within the defined searched range. A `Quick Search’ panel is also available on the homepage for the users to quickly retrieve the TSS or CP data of given gene name(s) with default parameters. The `Download’ utility allows users to download all raw data used in this database through a web interface. The download page shows a brief description of each data file for users to easily capture the file content. The Download section also provides a download link to a text file that includes detailed gene lists of 7193 orthologous groups in the 12 species. These orthologous groups were inferred using OrthoDB (67), which can be used to study the evolutionary dynamics of TSS landscape among orthologous genes during the evolution of yeast.

### Discussion

The YeasTSS database provides a user-friendly interface for users to visualize the transcription initiation landscape and promoter structures in yeast species. YeasTSS has already been demonstrated its usefulness as a tool to support research on transcription initiation processes in yeast. YeasTSS will be periodically updated when new TSS maps or functional genomic data are available. We plan to significantly extend the capabilities of the database to increase its usefulness and functionality. Specifically, we will (i) generate TSS maps using CAGE for more yeast species and for more *S. cerevisiae* strains, such as wild strains, wine and pathogenic strains; (ii) integrate more functional genomics data from *S. cerevisiae* as well as other yeast species; and (iii) provide a graph generating tool to produce figures of any genomic region of interest for publication purpose. The data and functionality are valuable for many studies, such as analysis of the regulatory mechanism of core promote usage, the evolutionary dynamic of promoter structures, identification of 5’ end boundaries of genes, improving the accuracy of genome annotation, prediction of novel genes, predictions of TFBSs and inference of gene regulatory network. Therefore, we are confident that YeasTSS will attract a broad audience and will become a popular resource of the research community in the field of genetics, genomics and molecular biology.

## Notes

**Database URL: http://yeastss.org/**
